# Sustainable removal of tetracycline and paracetamol from water using magnetic activated carbon derived from pine fruit waste

**DOI:** 10.1038/s41598-024-65656-3

**Published:** 2024-07-16

**Authors:** Farzad Hashemzadeh, Maryam Ariannezhad, Seyed Hamed Derakhshandeh

**Affiliations:** 1 Water and Wastewater Research Center, Water Research Institute, Tehran, Iran; 2 Department of Organic Chemistry, Faculty of Chemistry, Bu-Ali Sina University, Hamedan, 6517838683 Iran; 3 Department of Chemical Engineering, Faculty of Engineering, North Tehran Branch, Islamic Azad University, Tehran, Iran

**Keywords:** Environmental chemistry, Organic chemistry

## Abstract

This work presents highly porous magnetic activated carbon nanoparticles (MPFRC-A) derived from pine fruit residue. The MPFRC-A were produced through a three-step process: physical activation (carbonization temperature: 110–550 °C), chemical activation (H_2_SO_4_ (0.1 N, 96%)), and co-precipitation. These nanoparticles were then used to remove tetracycline (TC) and paracetamol (PC) from water. Functionalization with Fe_3_O_4_ nanoparticles on the surface of the pine fruit residue-derived activated carbon (PFRC-A) resulted in high saturation magnetization, allowing for separation from aqueous solution using an external magnet. The MPFRC-A adsorbent was characterized by Brunauer–Emmett–Teller (BET) analysis, Fourier-transform infrared spectroscopy (FTIR), scanning electron microscopy (SEM), X-ray diffraction (XRD), and Energy-dispersive X-ray spectroscopy (EDX) analyses, In the experimental section, the effects of various factors on the adsorption process were investigated, including pH, contact time, initial pollutant concentrations, adsorbent dosage, and temperature. Based on these investigations, adsorption isotherm models and kinetics were studied and determined. The results showed that MPFRC-A exhibited a large specific surface area (182.5 m^2^/g) and a high total pore volume (0.33 cm^3^/g). The maximum adsorption capacity was achieved at pH 6 and 5 for PC and TC drugs with an adsorbent dose of 400 mg and an initial concentration of 20 mg/L at 25 °C. The study revealed that the experimental data were well-fitted by the Langmuir isotherm model (R^2^ > 0.98), with maximum uptake capacities of 43.75 mg/g for TC and 41.7 mg/g for PC. Outcomes of the adsorption thermodynamics shows non-spontaneity of the reaction and the adsorption process by all adsorbents was endothermic.

## Introduction

Population growth and rapid industrialization are major contributors to global pollution problems^1^. Soil and water are directly affected by the sewage of different sectors such as chemical, pharmaceutical, agricultural, food and petrochemical industries, which creates severe risks for the environment^2–7^. Among emerging pollutants are pharmaceutical residues, constantly released into the hospital wastewater environment^8^. Studies over the past few decades have shown these compounds can contaminate aquatic systems^9,10^. One of the challenging issues is that the pharmaceutical residues in wastewater are not exterminate or removed in many cases. Among the systematically used medicines, Painkillers and antibiotics are a significant portion of the medicines humans and livestock consume^11–13^. For example, accordant with the World Health Organization (WHO), PC acts as a common nonsteroidal^14^, anti-inflammatory^15^, and antipyretic drug^16^ in the first line of pain treatment by inhibiting cellular mediators involved in the initiation of pain. Global PC production is estimated to reach $780 million by 2025, with China leading production at 64.4%^17^. Improper industrial effluent treatment allows PC to enter surface water, groundwater, and the natural environment^18^. On the other hand, cost-effective TC that shows lack any major adverse side effects^19^, used to treat bacterial infections, is also commonly used in animal feed^20^. A scientific study found a significan correlation between TC concentration in sewage and the number of antibiotic resistant bacteria^21,22^ If these types of bacteria enter the human body, they can pose a serious threat by limiting the effectiveness of medical prescription because they have become resistant to the TC^23,24^. Misuse, inappropriate treatment procedures, and overdoses of these medications lead to widespread detection of their residues in various surface water sources. This has extremely adverse impacts on many aspects of life.

Several conventional methods of water remediation such as photocatalytic degradation^25^, biodegradation^26^, nanofiltration^27^, electrochemical oxidation^28^, reverse osmosis^29^, etc. have been implied.. However, these methods often require complex technical setups and can be expensive. Additionally, they may generate harmful byproducts and exhibit lower effectiveness. Adsorption stands out as a preferred treatment method due to its cost-effectiveness, simplicity, high efficiency, and the availability of a wide range of adsorbents^30–32^. Carbonaceous materials derived from agricultural and food waste, such as wood sawdust^33^, coconut shells^34^, orange peel^35^, oak shell^36^, palm-kernel shells^37^, wood chips^38^, rice husk^39^, corn cobs^40^, seeds^41^, magnetic biochar^42^, etc. offer an eco-friendly approach to contaminant removal from effluents. These carbonaceous sources require activation to improve their adsorption capacity, which can be achieved through physical or chemical processes^43–45^. Physical activation of starting raw materials is carbonization of the residue waste and the consecutive activation at high temperature in a steam media. The carbonization of the already impregnated raw material with a chemical agent such as sulfuric acid, phosphoric acid, potassium hydroxide, zinc chloride, etc. is called chemical activation^46,47^. The adsorption of pharmaceutical compounds onto carbonaceous adsorbents occurs through binding with functional groups, particularly those containing oxygen. Chemical activation is often used to modify adsorbents with low specific surface areas, thereby enhancing their adsorption capacities^48^. Compared to physical activation, chemical activation offers advantages such as lower temperature requirements and a one-step process. Moreover, chemical activation process leads to higher yield and reduction in the mineral content. particularly, the bare Fe_3_O_4_ nanoparticles obtained significant attention because of their non-toxicity, bio compatibility, chemical stability, and hydrophilicity. Since they tend to aggregate, resulting in reduced surface area and adsorption potential. Therefore, designing the Fe_3_O_4_ nanoparticles is crucial to avoid this clustering drawback^49^.

In this work, a physical remedy (temperature: 110–550 °C) is used to transform the pine fruit residues into hard carbon powder. Afterwards, a chemical activation procedure using sulfuric acid (0.1 N) and pyrolysis temperature were completed to generate highly porous activated carbon. Finally, co-precipitation method was used to make magnetic carbon nanoparticles. To investigate the application of this adsorbent, two different types of pharmaceutical compounds, named TC and PC, were selected in an aquatic system, and the influences of important operational parameters, including solution pH, temperature, contact time, and dosage of the adsorbent, were evaluated. The properties of the adsorbent were explored via AAS, FTIR, XRD, BET, and SEM techniques and analyzed through kinetic, isotherm models, and thermodynamic studies.

## Characterization and discussion

### FT-IR spectroscopy

**Figure 1:**
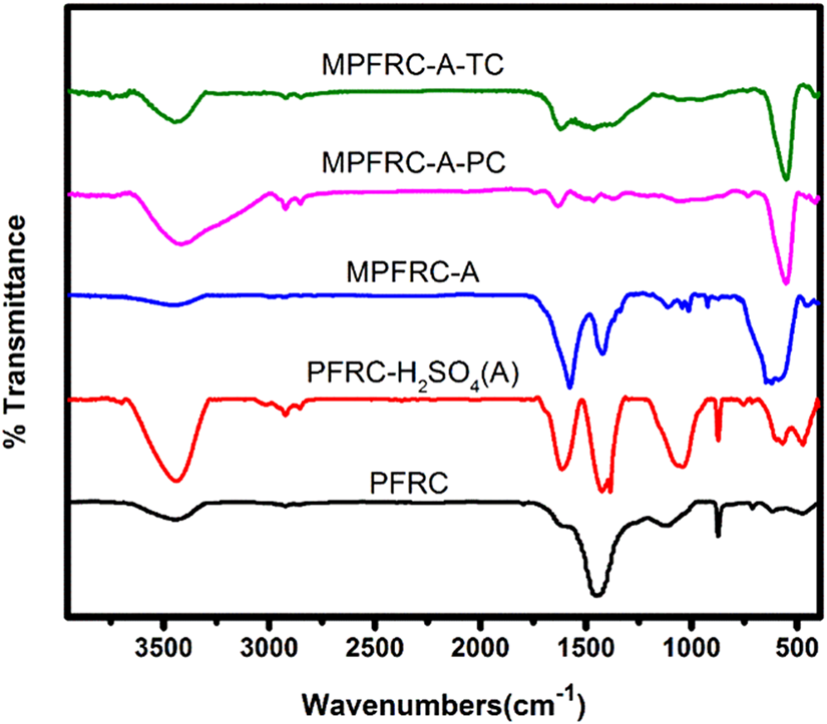
The FT-IR spectra each part of the preparation of adsorbent and adsorption process.

In this study, the chemical structure of each component involved in the preparation of the adsorbent and the adsorption process were investigated using FT-IR spectroscopy, as depicted in Fig. 1. The broad peak observed at 3439.45 cm⁻^1^ in spectrum 1a corresponds to the stretching vibration of OH bonds. This phenomenon is attributed to the presence of carboxylic acid and alcohol groups in cellulose or residual water^50^. Additionally, the peak observed in the 1614 cm⁻^1^ region is associated with the stretching vibration of C=O bonds, specifically for carbonyl and carboxylic acid groups. Furthermore, the band observed at 1449.5 cm⁻^1^ is related to the stretching vibration of C–C bonds due to the presence of aromatic rings^50^. After the pyrolysis process and subsequent activation, certain bonds were rearranged, leading to the formation of new functional groups on the surface of the PFRC-A adsorbent. Notably, the stretching vibration of S=O bonds at 1043 cm⁻^1^ provides clear evidence of sulfuric acid modification^51^. Finally, upon adsorption of TC and PC onto the adsorbent, the initial peak positions and intensities in the FTIR spectrum underwent changes, confirming the successful adsorption process^50^.

### BET measurements

**Figure 2:**
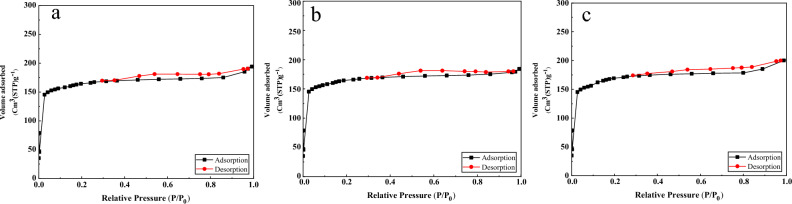
The N_2_ gas adsorption–desorption isotherm of (**a**) carbon, (**b**) m-carbon, and (**c**) carbon acid.

The BET method is employed to determine the specific surface area of materials. This technique relies on measuring the amount of nitrogen gas adsorbed within a relative pressure range of 0.1 to 1. Additionally, the pore size distribution is determined through the use of adsorption isotherms. In Fig. 2, the adsorption and desorption isotherms for two adsorbents are depicted. According to the IUPAC classification, these isotherms fall into Type IV (adsorption) and Type IV (desorption). Based on this classification, the pores exhibit a layered and sheet-like structure. Giving that Table 1, the specific surface area for carbon and m carbon adsorbents is equal to 195.5,182.5, and 224.6 m^2^/g, respectively. Also, in Table 1, the specifications of the absorbent surface are given. As can be seen, with chemical activation of the absorbent, the active sites have been improved and the available surface has increased.

**Table 1:** Results of the BET measurements of adsorbent.

Parameter	Carbon	M carbon	Carbon acid
a_s_ (m^2^/g)	195.5	182.5	224.6
V_m_ (cm^3^(STP) g^−1^)	96.46	94.66	109.69
V_p_ (cm^3^g^−1^)	0.42	0.33	0.61
r_p_ (nm)	5.06	4.21	6.86
a_p_ (m^2^/g)	117.24	115.44	130.02

### SEM analysis

**Figure 3:**
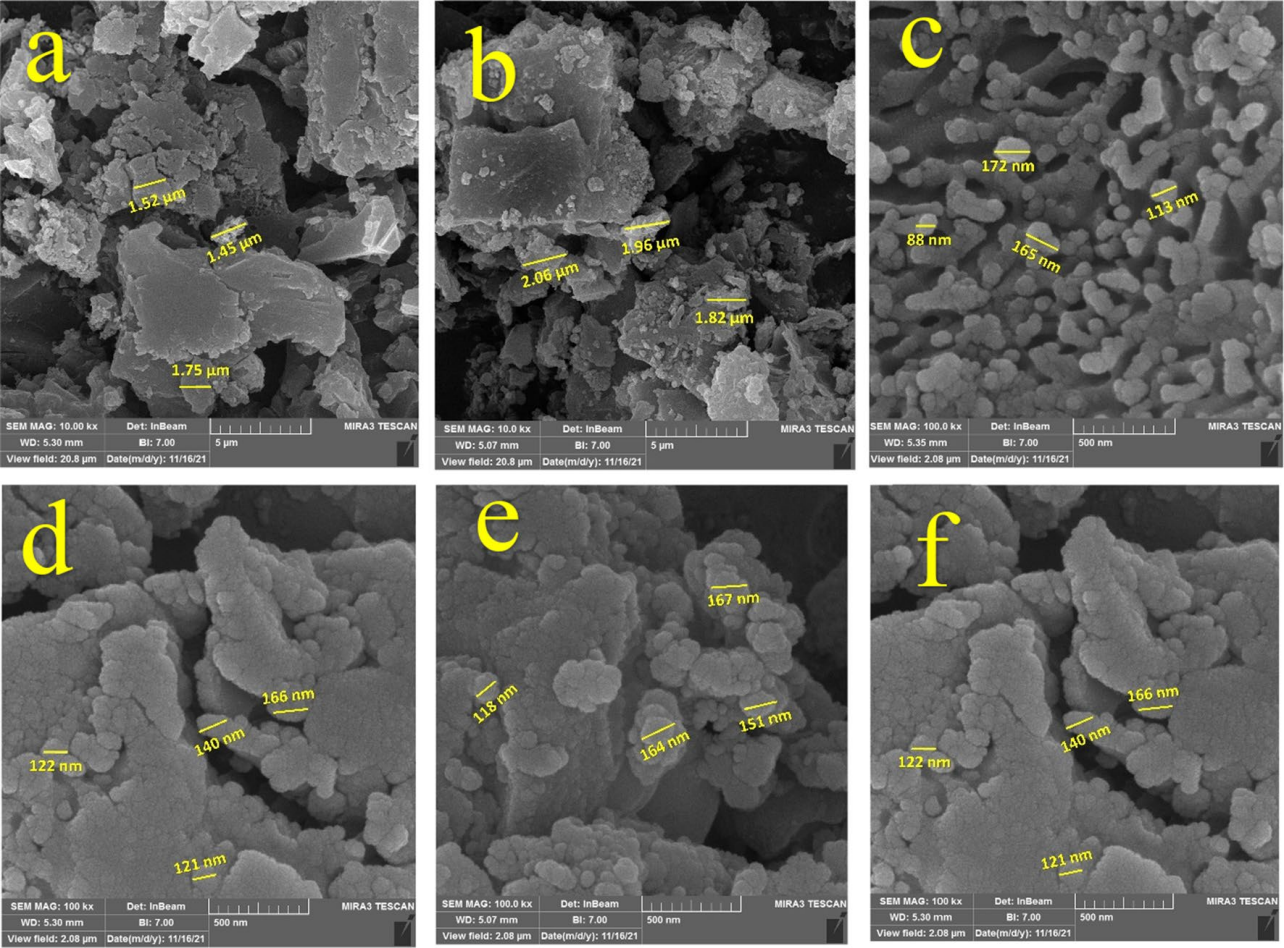
SEM images of (**a**,**b**) MPFRC-A, (**c**,**d**) MPFR-A/TC, (**e**,**f**) MPFRC-A/PC.

As seen in the SEM images of MPFRC-A before adsorption (Fig. 3), the adsorbent's surface appears relatively smooth, with varying pore sizes. However, after the adsorption of TC and PC, the surface becomes rougher, and the pore dimensions decrease. This reduction in pore size suggests the successful adsorption of the pharmaceutical compounds onto the adsorbent material's surface.

### XRD analysis

**Figure 4:**
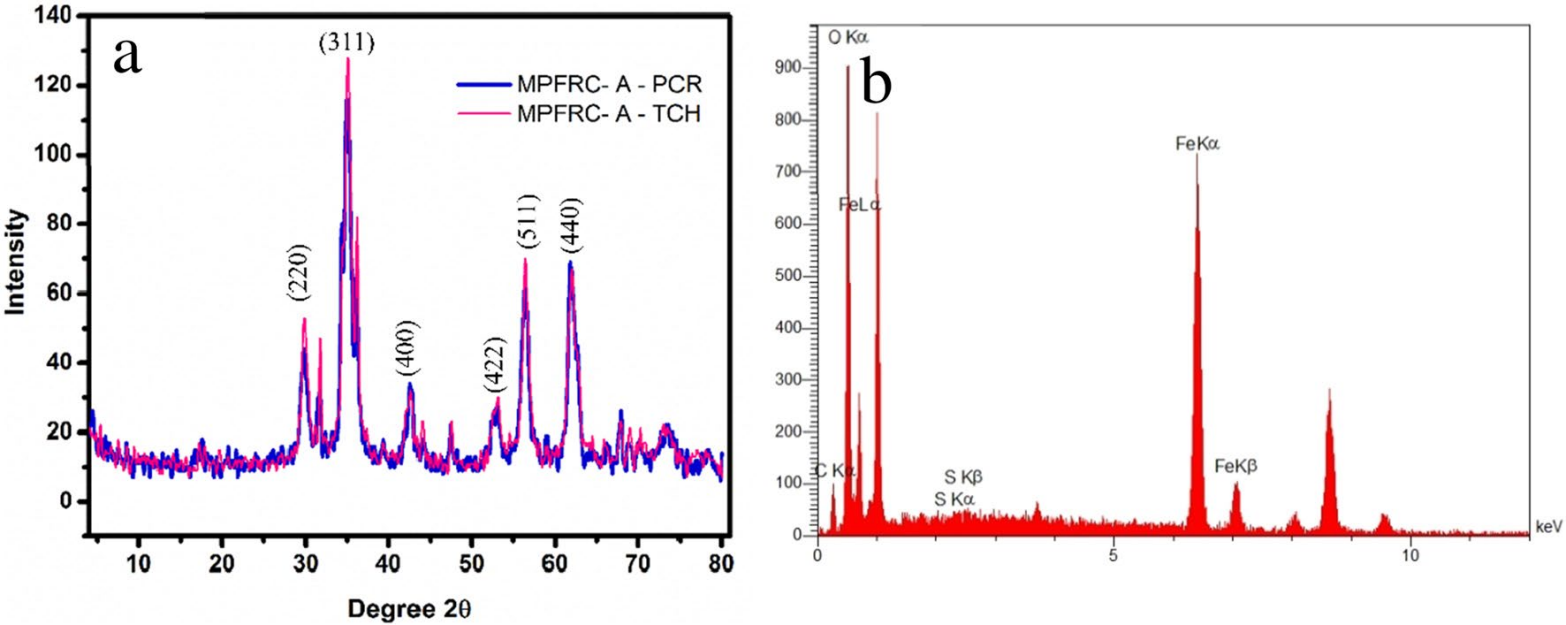
The (**a**) XRD patterns and (**b**) EDX analyses of MPFRC-A.

The observed patterns in several prior studies indicate the amorphous nature of MPFRC-A (2θ = 15.8° to 22.8°). This amorphous character is attributed to the presence of organic materials, such as hydroxyl and carboxyl groups^50^. Upon activation and thermal decomposition, the amorphous nature transforms due to the presence of elements like graphite, calcium, and silica, resulting in a semi-crystalline structure^50,51^. Typically, the activation of the adsorbent involves acid treatment and high temperatures, leading to the breakdown of functional groups within cellulose and hemicellulose, ultimately forming graphite. The sharp peaks observed at 2θ = 29.38° and 43.12° in Fig. 4a correspond to the presence of graphite, silica, and the amorphous carbon nature, primarily due to the low graphite content^50^. Also, XRD pattern of the synthesized MPFRC-A shows significant reflection peaks are found at 2θ = 30.09, 35.28, 43.02, 53.62, 56.91, and 62.73. When compared to the reference (JCPDS Card No. 19-0629), the peaks were confirmed to be magnetite nanoparticles. The peaks correspond to respective phase planes of (220), (311), (400), (422), (511), and (440)^42^. Also, the proposed structure for MPFRC-A was also confirmed by EDX analysis. As expected, the EDX measurement proves the presence of C, O, Fe, and S elements (Fig. 4b).

### VSM analysis

**Figure 5:**
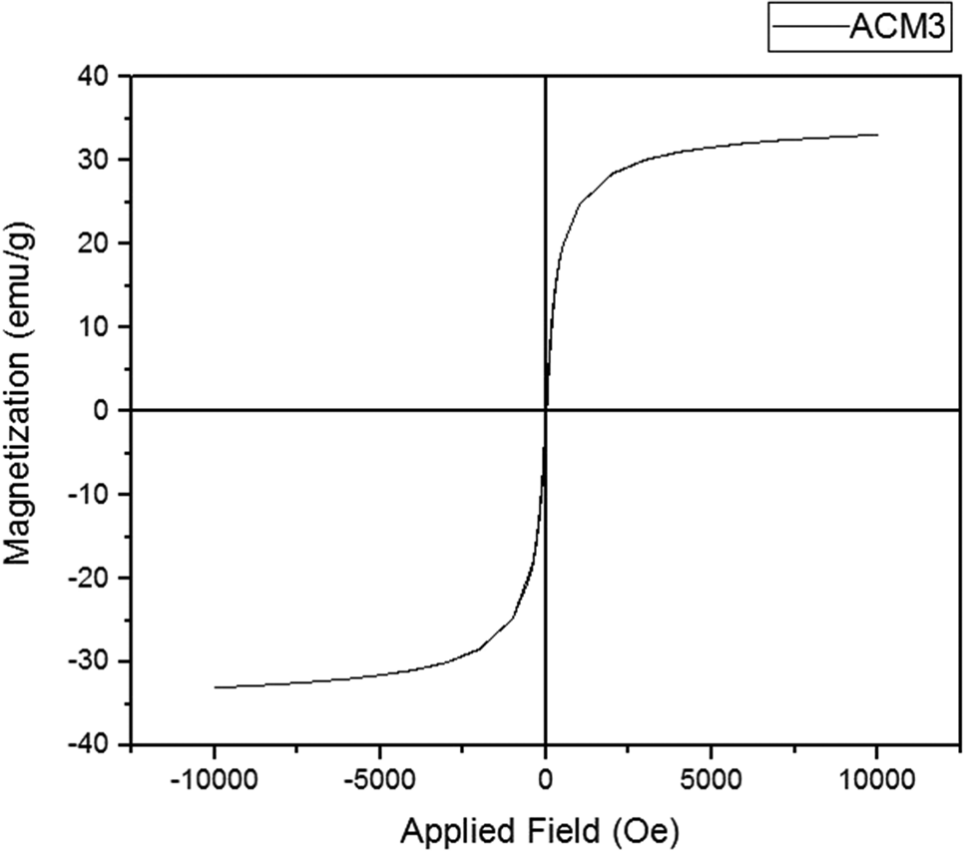
The VSM and of MPFRC-A.

The VSM analysis of MPFRC-A was performed in order to demonstrate the magnetic property (Fig. 5). As can be seen, the compound has magnetic property and shows a nice increase (35, emu/g), and probable reduction in the dipolar–dipolar interactions between the magnetic nanoparticles after mixing with activated carbon.

### pH

**Figure 6:**
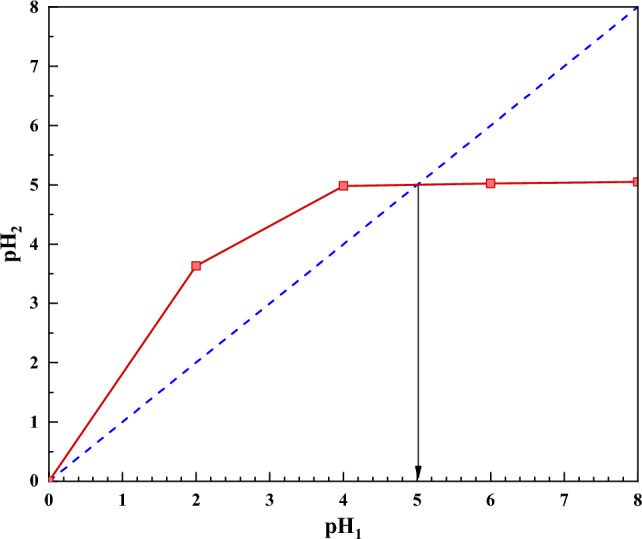
Isoelectric point of adsorbent by DRIFT method.

The pH of the point where the desired molecule or particle has no surface electric charge is called the pH_pzc_ or isoelectric point. Identifying the isoelectric point is essential for carrying out the adsorption process, because the creation of attractive or repulsive force between particles depends on this point. The determination of the isoelectric point of the adsorbents was determined by the drift method^52^. For this purpose, the pH of each of the four erlens, in which 20 mL of distilled water was poured into each one, and with the use of 0.1 M NaOH and 0.01 M HCl, was determined in one of pH 2, 4, 6 and 8 are adjusted with a pH meter. Then 0.5 g of adsorbent was added to them and the solution was stirred in a shaker for 24 h, the pH of the solution was measured again and the graph of secondary pH changes of the solutions was drawn according to the initial pH. The intersection of this graph with the line y = x shows the isoelectric point and was determined according to it. Figure 6 shows the results of secondary pH in terms of initial pH. According to the obtained results, the isoelectric point of the adsorbent was found to be equal to 5. The surface of the adsorbent at a pH higher than this value has a negative charge, and the surface of the adsorbent at a pH lower than this value has a positive charge.

**Figure 7:**
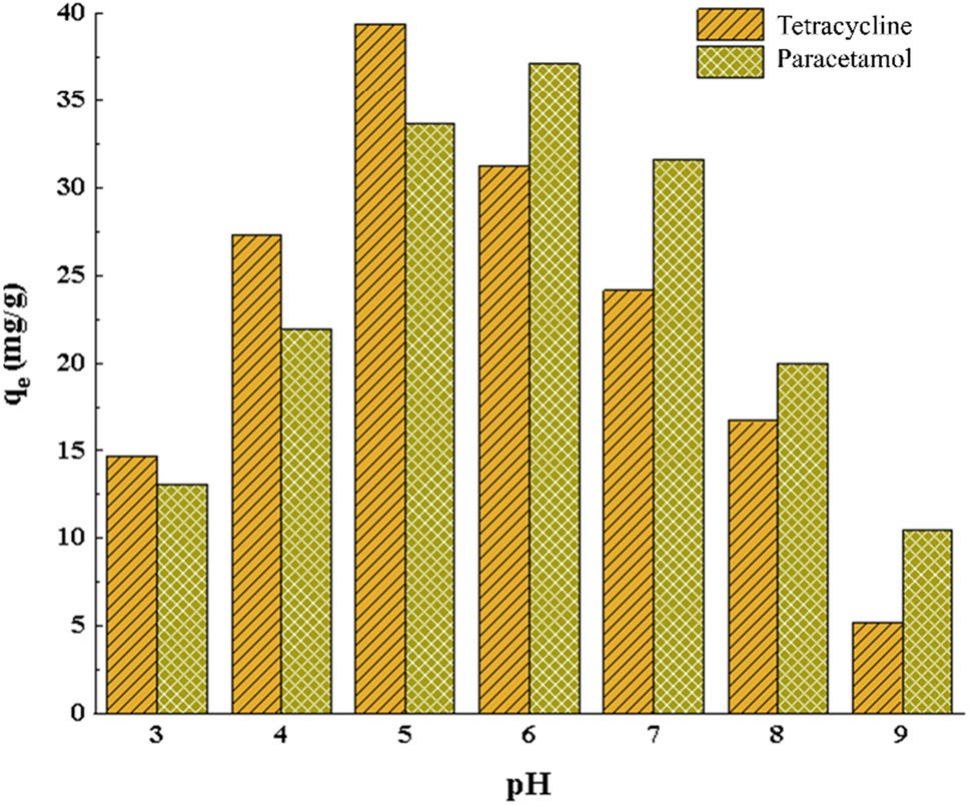
The effect of pH on the adsorption capacity of PC and TC (Initial concentration 20 mg/L, m = 0.4 g, V = 35 ml and T = 25 °C).

The pH of a solution is one of the most influential factors in the adsorption process and the mechanism of electrostatic forces. To determine the optimal pH for the adsorption of PC and TC, each drug was individually tested at a concentration of 20 mg/L, using 0.4 g/L of the adsorbent within the pH range of 3 to 8. Considering the pk_a_ values of the drugs and the isoelectric point of the investigated adsorbent (which is 5), we observe that at pH values below the isoelectric point, the surface charge of the adsorbent becomes positive, while at pH values above the isoelectric point, the surface charge becomes negative. The results of the pH effect on adsorption capacity are illustrated in Fig. 7. For PC pk_a_ is equal to 9.51. Also, the pH_pzc_ of the adsorbent is equal to 5, as a result, at pHs greater than 9, it is negative with both pollutant and adsorbent, and at pH less than 5, it is positive with both pollutant and adsorbent, as a result, in this pH pollutant and adsorbent repel each other and the adsorption capacity is low. At pH between 5 and 9 pollutant and adsorbent are not synonymous and attract each other. As a result, at pH equal to 6, the highest adsorption capacity is observed. TC has three pk_a_ of 3.30, 7.68 and 9.68. TC is an organic molecule whose protonated or deprotonated form depends on the pH of the solution. TC appears at pH less than 3.3 due to the protonation of amine groups in a cationic form with two positive charges, which is why According to the surface charge of the adsorbent, the pollutant and the adsorbent both have a positive charge and repel each other. At pH 3–7, one end of TC has a positive charge and the other end has a negative charge, and the highest amount of removal occurs at this pH. And finally, at a pH higher than 9, due to the loss of a proton from the carboxylic group in the structure of the antibiotic, it appears in a completely anionic form with two negative charges, at this pH, according to the surface charge of the adsorbent, pollutant and adsorbent of each the two have a negative charge and repel each other. As a result, the highest adsorption capacity occurs at pH 6 and 5 for PC and TC drugs. Considering that pH change affects the adsorption capacity of two drugs, it can be said that electrostatic forces are involved in the adsorption process.

### Dosage of adsorbent

**Figure 8:**
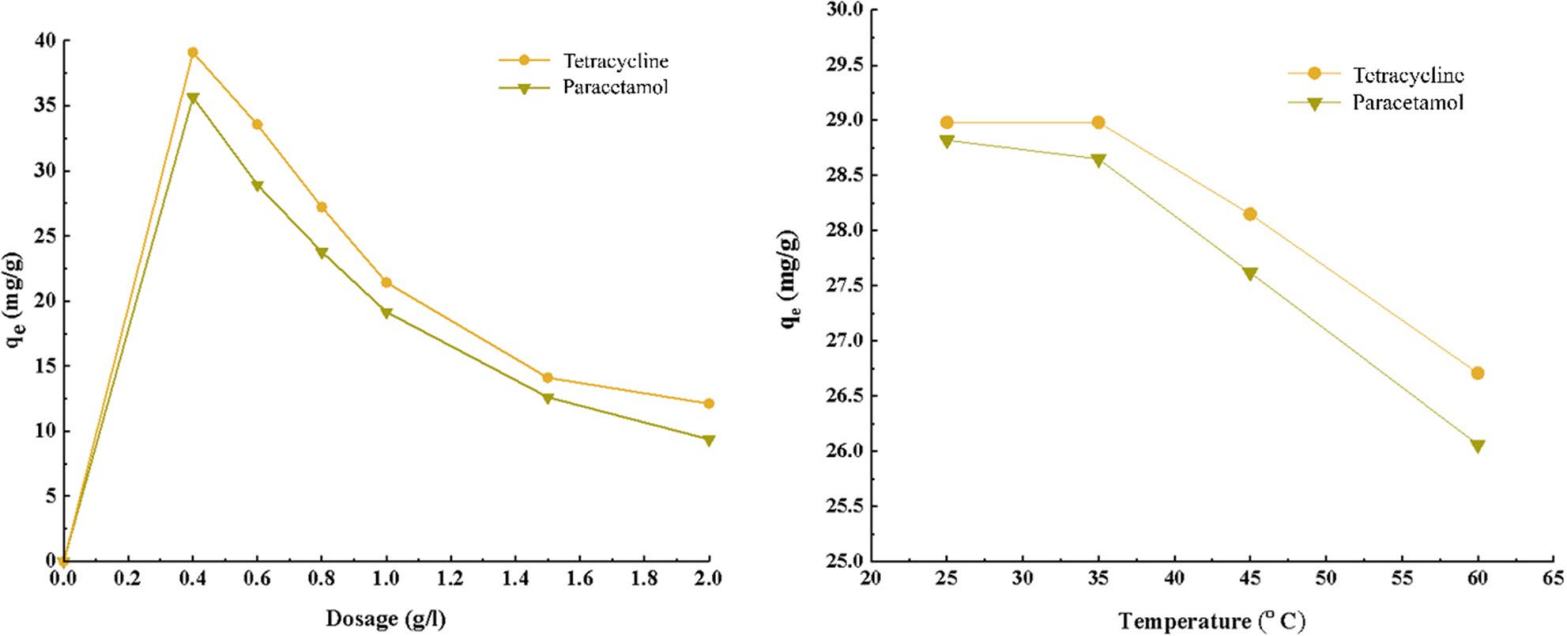
(**a**) The effect of adsorbent dosage on absorption capacity of PC and TC (initial concentration of 20 mg/l, pH equal to 4 and 5, duration of 120 min and ambient temperature), (**b**) The effect of temperature on absorption rate of PC and TC (initial concentration 20 mg/l, pH equal to 4 and 5, duration of 120 min and ambient temperature, amount of adsorbent 0.4 g).

To determine the optimal amount of adsorbent, different amounts of adsorbent 0.4, 0.6, 0.8, 1, 1.5 and 2 g/L of solution with an initial concentration of 20 mg/L, pH equal to 4 and 5, and a duration of 120 min were investigated. As can be seen in Fig. 8a, the adsorption capacity raises with an increase in the adsorbent amount from 0 to 0.4 g/L due to the availability of unsaturated adsorption sites. However, by increasing the adsorbent amount from 0.4 to 2 g/L, the adsorption capacity for both drugs decreases due to the agglomeration and clumping of the adsorbent, reducing the available active surface area. Therefore, an adsorbent amount of 0.4 g/L was considered optimal for further experiments.

Also, with the increase in temperature, the speed of movement of drug molecules in the solution increases (Fig. 8b). As a result, the energy of the random collision speed increases and causes the absorption capacity to decrease. Therefore, an increase in temperature breaks the bonds created between the absorbent and the pollutant and disposal occurs. According to the Fig. 8, the maximum absorption capacity is related to the ambient temperature, so the temperature of 25 degrees Celsius was chosen to continue the experiments.

### Thermodynamic of adsorption

**Figure 9:**
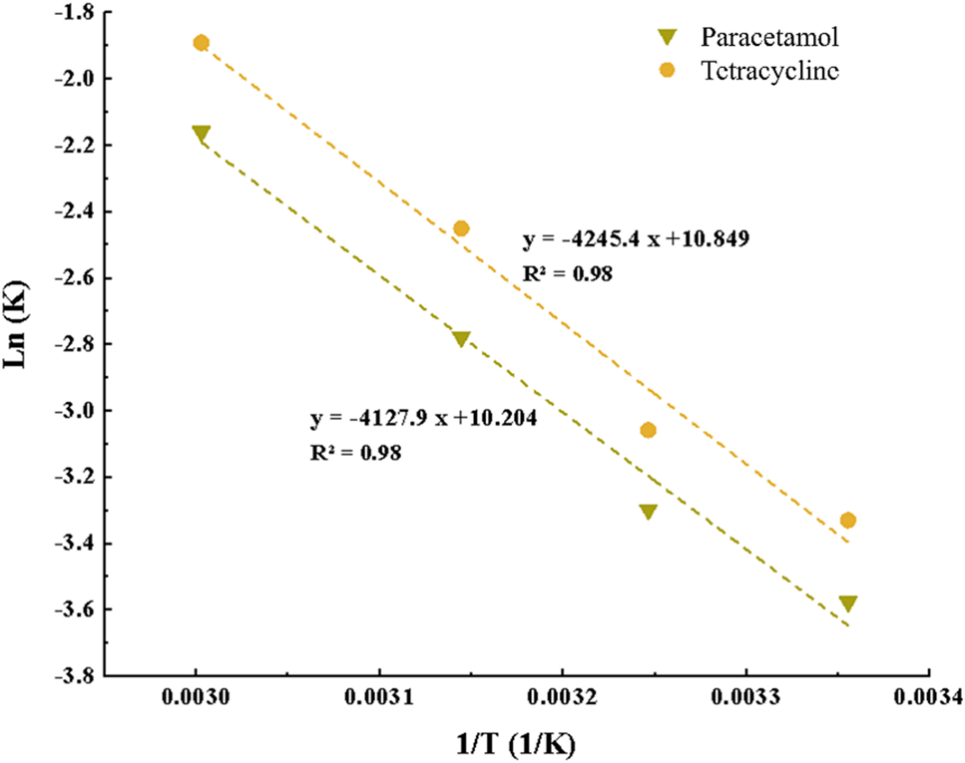
Van't Hoff curve.

Enthalpy and entropy may be calculated from the Van't Hoff curve by researching how temperature affects adsorption capacity (Fig. 9)^53,54^.

Table 2 lists the outcomes of the adsorption thermodynamics drawn from the Van't Hoff diagram. Table 2 shows that ∆G° was positive, indicates the non-spontaneity of the reaction. Throughout the procedure, irregularities in terms of the positive values of ∆S° increased. Based on the value of ∆H°, which was positive, the adsorption process by all adsorbents was endothermic. Moreover, based on the references, and values of ∆H°, which were elder than − 20 kJ, it can be concluded that the adsorption band was physical^55,56^.

**Table 2:** Thermodynamic parameters of the adsorption process.

Drugs	∆S (J/mol/K)	∆H (KJ/mol)	∆G (KJ/mol)			
			298 (°K)	308 (°K)	318 (°K)	333 (°K)
PC	84.83	35.29	10.01	9.16	8.31	7.04
TC	90.19	34.31	7.43	6.53	5.62	4.27

### Effect of contact time and kinetics

In our study, we investigated the adsorption kinetics of TC and PC to understand the impact of contact time on their adsorption behavior. The initial solutions were prepared with a concentration of 20 mg/L, and 0.4 g/L of each adsorbent was added. To ensure that the adsorption process reached equilibrium, the samples were agitated for 2 h in a shaker operating at 250 rpm and maintained at a temperature of 25 °C. The results of this investigation shed light on the dynamic behavior of TC and PC adsorption, providing valuable insights for environmental and pharmaceutical applications.

The obtained data were fitted to pseudo-first-order, pseudo-second-order, and intraparticle diffusion models, with the results summarized in Table 3. Based on the correlation coefficient (R^2^) for each model, the pseudo-second-order model is deemed acceptable for the adsorption of TC and PC. The pseudo-second-order kinetic model operates under the assumption that the rate-limiting step involves ion exchange. Additionally, the rate constants for the adsorption of TC and PC are estimated to be 0.116 g/mg.min and 0.096 g/mg.min, respectively. The investigation of intraparticle diffusion kinetics provides valuable insights into the transport of solute species within porous adsorbents. Notably, the rate constant for intraparticle diffusion falls within the range of 3.21 to 3.78, while the constant C signifies the boundary layer thickness and characterizes the external mass transfer process. Furthermore, the pronounced increase in adsorption capacity during the initial stages of the process is attributed to the phenomenon of rapid mass transfer.

**Table 3:** Constants and coefficients related to adsorption kinetic models.

Model	Parameter	PC	TC
Pseudo-first-order	*q*_*e*_ (mg g^−1^)	36.2	40.32
	*K*_1_ (min^−1^)	0.04036	0.047
	*R*^2^	0.98	0.98
Pseudo-second-order	*q*_*e*_ (mg g^−1^)	44.41	47.94
	*K*_2_*/1*0^−2^ (g/(mg min))	0.096	0.116
	*R*^2^	0.99	0.99
Intraparticle diffusion	*C* (mg/g)	3.21	3.78
	*k*_*ipd*_ (mg.min^−0.5^ g^−1^)	3.46	5.11
	*R*^2^	0.91	0.88
Elovich	*α*	0.26	0.20
	*β*	8.34	3.42
	*R*^2^	0.76	0.78

**Figure 10:**
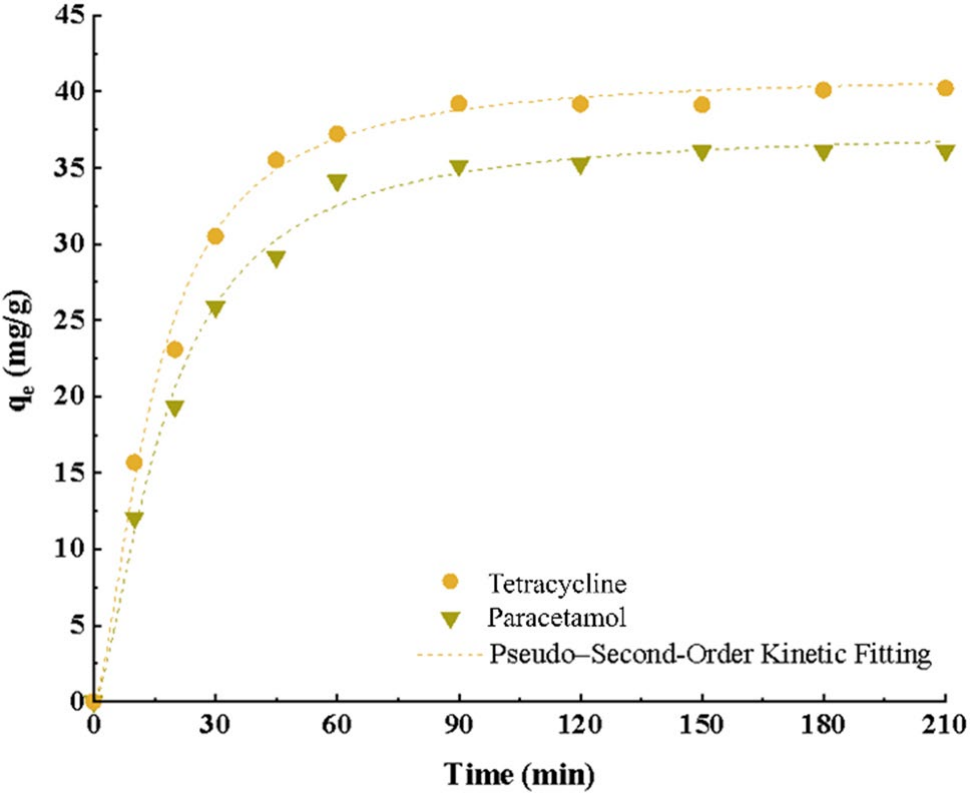
The pseudo-second-order kinetic fitting for adsorption of PC and TC (Initial concentration 20 mg/L, pH = 5 and 6, T = 25 °C).

As shown in Fig. 10, in the early times, the slope of the graph is very steep (the rapid phase of external mass transfer) and as the equilibrium conditions are approached, the speed of the adsorption process slows down (the slope of the graph decreases and reaches zero) until the adsorbent does not have more pollutant adsorption power and the adsorption graph is fixed^57,58^. Also, after about 30 min, the adsorption graph stabilizes and the adsorption reaches equilibrium, however, to ensure the achievement of complete equilibrium, the studied time period was continued up to 120 min.

### Adsorption isotherm

In the context of adsorption processes, quantifying the removal efficiency of pollutants from aqueous solutions is of paramount importance. To achieve this, we employ adsorption isotherm models, which explore the surface properties and affinity of the adsorbent toward the adsorption process. In this study, we investigated the adsorption behavior of TC and PC using the Langmuir, Freundlich, and Temkin isotherm models, as well as the three-parameter Redlich–Peterson model. The Langmuir model had the most overlap with the experimental results of TC and PC adsorption. This model describes the adsorption process in terms of a monolayer at equilibrium. The maximum adsorption capacity (q_m_) represents the adsorption capacity of a single layer. For TC and PC, the Langmuir model yielded high R^2^ = 0.99. The Langmuir constants K_L_ characterizes the adsorbent’s affinity for the pollutant. The calculated values of q_m_ for TC and PC were 43.75 mg/g and 41.70 mg/g, respectively. By comparing the experimental data with the Langmuir isotherm, we observed excellent agreement between the model predictions and the measured adsorption capacities (as shown in Table 4). In summary, the Langmuir isotherm provides a robust framework for understanding the adsorption behavior of TC and PC, offering valuable insights for environmental remediation and pharmaceutical wastewater treatment.

**Table 4:** Constants and coefficients of adsorption isotherm models for PC and TC.

Model	Parameter	PC	TC
Langmuir	*q*_*max*_ (mg.g^−1^)	41.7	43.75
	*K*_*L*_ (L.mg^−1^)	0.69	0.84
	*R*^2^	0.98	0.98
Freundlich	*K*_*F*_ (mg^1−n^ L^n^ g^−1^)	17.1	16.97
	*n*	2.75	2.69
	*R*^2^	0.91	0.95
Temkin	*B*_*T*_ (Kj.mol^−1^)	9.41	8.62
	*A*_*T*_ (L.mg^−1)^	6.55	8.91
	*R*^2^	0.91	0.97
Redlich–Peterson	*K*_*RP*_ (L.mg^−1^)	25.74	39.56
	_*RP*_*α*_(L.mg_^−1^_)_	0.44	1.08
	_*Rp*_*β*	1.12	0.93
	*R*^2^	0.98	0.98
Hill	*q*_*H*_ (mg.g^−1^)	38.41	41.68
	*K*_*H*_	1.029	1.171
	*n*_*H*_	1.402	1.004
	*R*^2^	0.98	0.98

The Freundlich isotherm, which characterizes heterogeneous surface adsorption, has been employed to investigate the interaction between the adsorbent and the pharmaceutical compounds. As shown in Table 3, the R^2^ for TC and PC adsorption using the Freundlich model is 0.95 and 0.91, respectively. Notably, these values are lower than those obtained from the Langmuir isotherm. This discrepancy underscores the limitations of the Freundlich model in describing the adsorption behavior of the aforementioned drugs. The parameter n in the Freundlich isotherm reflects the favorability of the adsorption process. For the range of n values between 0 and 10, the experimental data in Table 4 confirm favorable adsorption behavior. In summary, while the Freundlich isotherm provides insights into surface heterogeneity, the Langmuir model remains a more robust choice for describing the adsorption performance of TC and PC.

The Temkin isotherm model postulates that the heat of adsorption decreases linearly with coverage. Additionally, it assumes that the adsorption process is characterized by a uniform distribution of cohesive binding energies. According to the data presented in Table 4, the heat of adsorption follows a linear trend with decreasing surface coverage. The parameter A_T_ represents the equilibrium binding energy, corresponding to the maximum bond energy, while B_T_ pertains to the heat of adsorption. A low value of B_T_ indicates weak interactions between the adsorbate molecules and the adsorbent surface.

Hill's isotherm model, in this model, it is a cooperative phenomenon where the ligand is attached in one place on the macromolecules. In this case, it may affect different binding sites on the same macromolecules. q_H_ is the adsorption capacity of the Hill isotherm, n_H_ is the Hill binding interaction coefficient, and K_H_ is the Hill constant.

**Figure 11:**
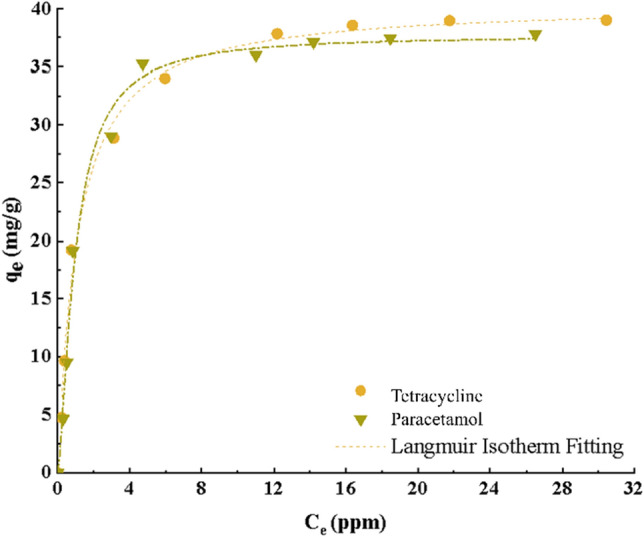
The Langmuir isotherm model fitting for the adsorption of PC and TC (Time = 120 min, pH = 5 and 6, T = 25 °C).

Among the three-parameter adsorption isotherm models, the Redlich–Peterson model is frequently employed for liquid-phase adsorption of organic compounds. Based on the obtained correlation coefficients, both the Langmuir isotherm model and the Redlich–Peterson model outperformed the two-parameter models. This observation suggests that the adsorbent surface becomes more closed during surface adsorption, leading to a reduction in the influence of external factors on drug adsorption. According to the results, the adsorption of pharmaceutical pollutants on the researched adsorbent is of single layer type. The adsorbent structure is homogeneous and has the same adsorption energy. Also, adsorption has been done physically. Considering that the value of β in the Redlich–Peterson equation is close to 1, the Langmuir model is more consistent with the experimental equilibrium data. Figure 11 shows the fit of the Langmuir model curve.

### Mechanism

The adsorption mechanism is evaluated according to the adsorbent properties, such as pores, functional groups, surface area, and also pH value. It is suggested dominant interactions could be electrostatic attraction, and Ion exchange between functional groups of Tetracycline and Paracetamol, and the protonated surface area of adsorbents in acidic media. Also, bonding between pharmaceutical compounds and carbonaceous material on the surface could be occurred in electron donor–acceptor hydrogen, and π-hydrogen interaction^59^.

### Recovery

**Figure 12:**
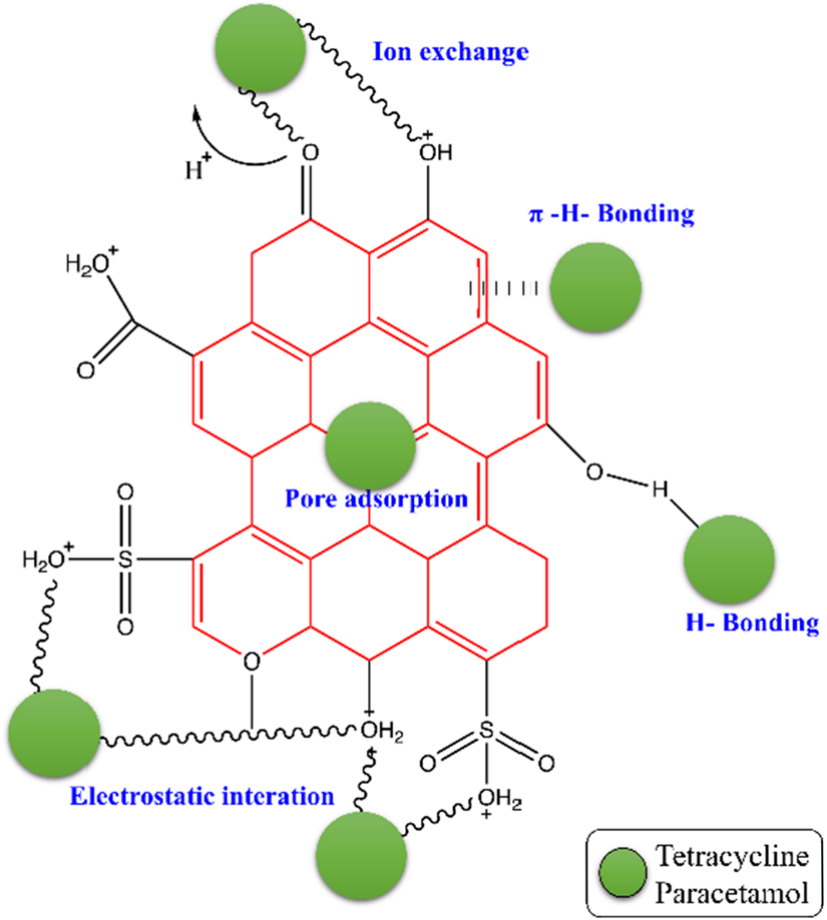
The adsorption mechanism of TC and PC by MPFRC-A.

**Figure 13:**
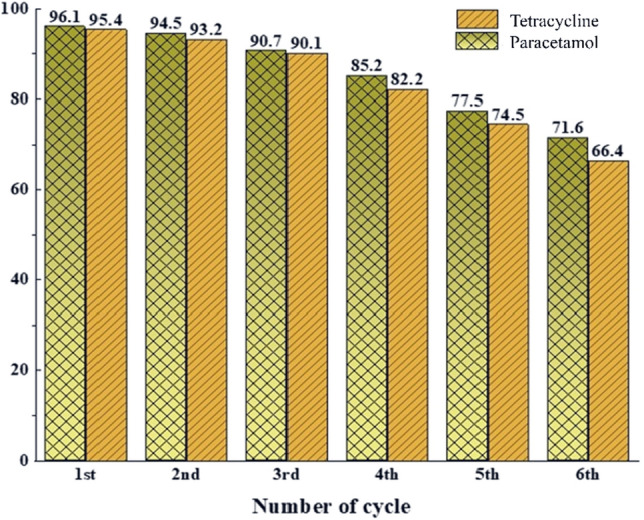
Reusability of MPFRC-A.

Adsorbent recovery for reuse is important from an economic perspective (Fig. 12). Although the price of synthesized adsorbents is higher, but due to their high absorption capacity, they can be used in smaller amounts than other mentioned adsorbents, which will bring a higher economic value. To recover the adsorbents, after each use, we add 5 cc of ethanol to the adsorbent, it is placed on the shaker for 20 min, then it is separated with a magnet, and in the next step, 5 cc of the buffer prepared in the ratio (1:10) is added to it and placed on the shaker for 20 min and finally separated by a magnet. According to Fig. 12, the synthesized adsorbent for PC and TC drugs can be used 6 times with a removal efficiency of 71.6 and 66.4% (Fig. 13).

### Plant collection statement

We confirm that our experimental research and field studies on plants, both cultivated and wild, strictly adhere to the relevant institutional, national, and international guidelines and legislation. The collection of plant material was conducted in accordance with these guidelines, ensuring ethical and responsible practices throughout our study.

## Conclusion

The present work demonstrates the successful removal of TC and PC from aqueous solutions using activated carbon produced from pine cone waste. The carbon’s activity was evaluated through experiments involving H_2_SO_4_-treated aqueous samples. Several key parameters, including pH, initial concentration, contact time, temperature, and adsorbent dosage, were systematically investigated. The maximum adsorption capacity of the adsorbent was achieved under the following conditions: pH = 4 and 6, adsorbent dose = 400 mg and initial concentration 20 mg/L at 25 °C. For TC, the maximum adsorption capacity reached 43.75 mg/g, while for PC, it was 41.7 mg/g. The experimental data closely aligned with the Langmuir isotherm model (with an R^2^ > 0.98), indicating that the adsorption occurred on a heterogeneous surface. Notably, the adsorbent can be regenerated through sequential washing with ethanol and buffer solution, allowing for reuse. In summary, utilizing activated carbon from pine cone waste offers several advantages, including environmental compatibility, reasonable adsorption capacity, cost-effectiveness, and non-toxicity. These natural adsorbents, with their substantial surface area, hold significant promise for pharmaceutical compound removal from aqueous solutions, contributing to both economic and environmentally friendly practices in industrial settings.

## Materials and methods

Iron **(**III**)** chloride hexahydrate, Iron (III) chloride tetrahydrate, Sulfuric acid 96%, Acetic acid (glacial) 100%, PC, Aqueous ammonia (37%), TC hydrochloride, Ethanol 96%, orto-Phosphoric acid 85%, Boric acid were purchased from Merk company. Pine fruit residues were selected and collected from Chitgar forest, Tehran Province, Iran. Distilled water was purchased from Tamad kala, Iran.

### Preparation of MPFRC-A

In the first step, activated carbon was synthesized using pine fruit waste. The pine residue was washed with distilled water well to remove the pollen and dried under the sun for 5 days, then put in the furnace for 8 h at a temperature of 105–103 °C until it dried completely, after that for the complete carbonization, the dried shells were placed in the furnace at 550°C for 20 min. afterwards, the samples were taken out of the oven and let them cooled down, and then they were pounded by a mortar until they were completely powdered. In order to homogenize the parts, they were sieved by a laboratory sieve and finally, the resulting powder was kept in a desiccator^50^.

In the second step, in order to generate more porosity in the obtained carbon from the previous step, it was soaked with H_2_SO_4_ (0.1 N, 96%) with a glass stirrer and put in furnace at 110 °C for 24 h (soaking). Then, it washed with distilled water until reaching a stable and neutral pH, and this step was repeated several times in such a way that the above solution is poured into Falcon tubes, distilled water was added to it and mixed with a vortex machine. Finally, for complete separation, it was placed in a centrifuge at 5000 rpm for 20 min, and after neutralization, it was dried in an oven at a temperature of 103 °C, and after cooling it was kept in a desiccator at room temperature^50^.

In the third step, Co-precipitation method was used to make magnetic carbon nanoparticles. For this purpose, FeCl_3_ and FeCl_2_ were dissolved in a glass flask containing 100 ml of deionized water with a molar ratio (2:1) and 1 g of carbon obtained from the previous step was added to the above solution. The color of the solution at this stage was orange. Then aqueous ammonia (37%) was slowly added to the balloon until the solution turned completely black and its pH reached to 10. The solution was stirred at 80 °C for 2 h. This step of synthesis was done under nitrogen gas. Then, the synthesized magnetic carbon nanoparticles were separated from the balloon with an external magnet, washed and dried in a desiccator at room temperature.

### Adsorption experiments

Several parameters affect the phenomenon of adsorption such as initial concentration of TC and PC solutions, contact time, pH, and mass of MPFRC-A. Adsorption experiments were followed by using 400 mg MPFR-AS for all solutions (35 ml) with concentrations of 10–50 mg / l for TC and PC. The glass Erlenmeyer flakes were stirred at 200 rpm for 2 h in the ambient temperature. Afterwards, the contents of the Erlenmeyer flask were separated by an external magnet. The separated liquids were analyzed via the spectrophotometer (DR6000). The obtained results reveal that the initial concentration of the pharmaceutical residue is one of the most noticeable factors impacting the adsorption process. Generally, the data observed in Table 5 showed that a slight increase in the initial concentration of the pharmaceutical residue leads to an increase in removal percentages as well. However, with a limited amount of adsorbent, there is a risk of the adsorbent becoming saturated at high concentrations. Therefore, we selected 20 mg/l as the optimum concentration. Subsequently, the pH, adsorbent amount, adsorbent dosage, and contact time were investigated to achieve reasonable data for studying adsorption kinetics and adsorption isotherms.

**Table 5:** Adsorption percentage in 10–50 (mg/ l) concentrations of TC and PC by MPFRC-A (reported by the spectrophotometer DR6000).

Conct. of TC or PC (mg/ l)	%Adsp TC	%Adsp PC
10	78.16	73.11
20	83.15	81.18
30	85.21	88.14
40	89.13	91.17
50	91.14	91.17

The rate of TC and PC uptake on MPFRC-A as adsorption capacity $q$ (mg/g) was obtained according to below equation.

$$q=\frac{\left({C}_{0}-{C}_{e}\right)\times V}{m}$$

where C_0_ and Ce stand for the initial and equilibrium concentration respectively. V and m are defined the volume of the solution (L) and the weight of the adsorbent (g).

## Data Availability

All data generated or analysed during this study are included in this published article.
